# Robust numerical evaluation of circular dichroism from chiral medium/nanostructure coupled systems using the finite-element method

**DOI:** 10.1038/s41598-018-26815-5

**Published:** 2018-05-30

**Authors:** Seojoo Lee, Ji-Hun Kang, SeokJae Yoo, Q-Han Park

**Affiliations:** 1 Department of Physics, Korea University, Seoul, 02841 Korea; 2 Department of Physics and Astronomy, Seoul National University, Seoul, 08826 Korea

## Abstract

It has been demonstrated that circular dichroism (CD) signals from chiral molecules can be boosted by plasmonic nanostructures inducing strong local electromagnetic fields. To optimize nanostructures to improve CD enhancement, numerical simulations such as the finite element method (FEM) have been widely adopted. However, FEM calculations for CD have been frequently hampered by unwanted numerical artifacts due to improperly discretizing problem spaces. Here, we introduce a new meshing rule for FEM that provides CD simulations with superior numerical accuracy. We show that unwanted numerical artifacts can be suppressed by implementing the mirror-symmetric mesh configuration that generates identical numerical artifacts in the two-opposite circularly polarized waves, which cancel each other out in the final CD result. By applying our meshing scheme, we demonstrate a nanostructure/chiral molecule coupled system from which the CD signal is significantly enhanced. Since our meshing scheme addresses the previously unresolved issue of discriminating between very small CD signals and numerical errors, it can be directly applied to numerical simulations featuring natural chiral molecules which have intrinsically weak chiroptical responses.

## Introduction

Circular dichroism (CD) can be found in various natural substances including molecules and crystals and is of particular importance to the study of biology and chemistry^1,2^. Because the interaction of incident light with chiral molecules is generally quite weak, nanostructure-molecule coupling has been proposed as a way to enhance CD by inducing strong near fields that boost the optical helicity density of the incident light^3–17^. Various numerical schemes^18–20^ including the finite element method (FEM)^12,21–23^ have been widely adopted to estimate enhancements in optical helicity density and optimize the nanostructures. However, for CD calculations, FEM has been used without in-depth consideration of meshing, i.e., the discretization of the problem space, so possible numerical artifacts arising from meshing have not been taken into account. Since CD calculation demands a difference between two signals, the left- and right-circularly polarized lights, numerical artifacts can be cumulative when not consistently generated by the two different polarizations.

**Figure 1:**
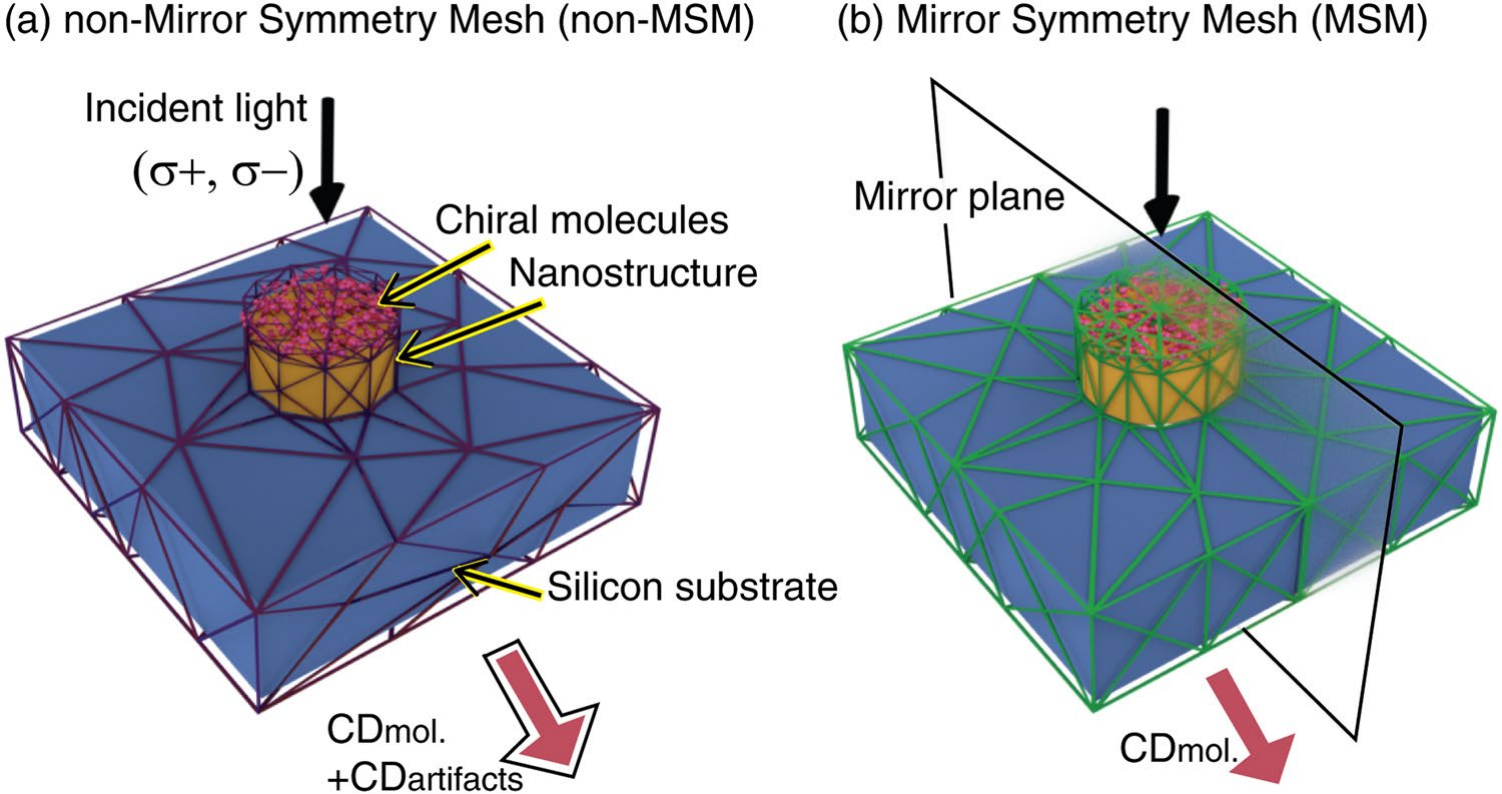
Schematics of non-mirror symmetric mesh (non-MSM) and mirror symmetric mesh (MSM). Discretized chiral molecule/gold nanostructure coupled system by (**a**) non-MSM and (**b**) MSM. MSM has a mirror symmetry with respect to the mirror plane.

In this paper, we introduce a new meshing scheme, mirror symmetric mesh (MSM), that possesses a mirror symmetry with the mirror plane containing the light wavevector. Such a MSM provide a symmetric mesh configuration against the left- and right-circularly polarized lights. MSM can be applied to FEM for CD calculations which features suppressed numerical artifacts. Non-negligible numerical artifacts arise especially when the chiral medium is coupled with nanostructures, and they become noticeably stronger when the implementation of a periodic structure is required. We show that, compared to the usual non-mirror symmetric mesh (non-MSM) shown in Fig. 1(a), our MSM scheme shown in Fig. 1(b) provides superior numerical accuracy by eliminating the unwanted numerical artifacts in such a way that they cancel each other out in the final CD calculations. Our meshing scheme was tested on the calculation of the CD of a chiral molecule/nanodisk coupled system, and we show that our scheme exhibits much faster numerical convergence regarding finite-element grid size than the usual meshing schemes, which require much finer grids and longer computation times to achieve convergence.

## Results

### Numerical CD artifacts from broken mirror-symmetric mesh

**Figure 2:**
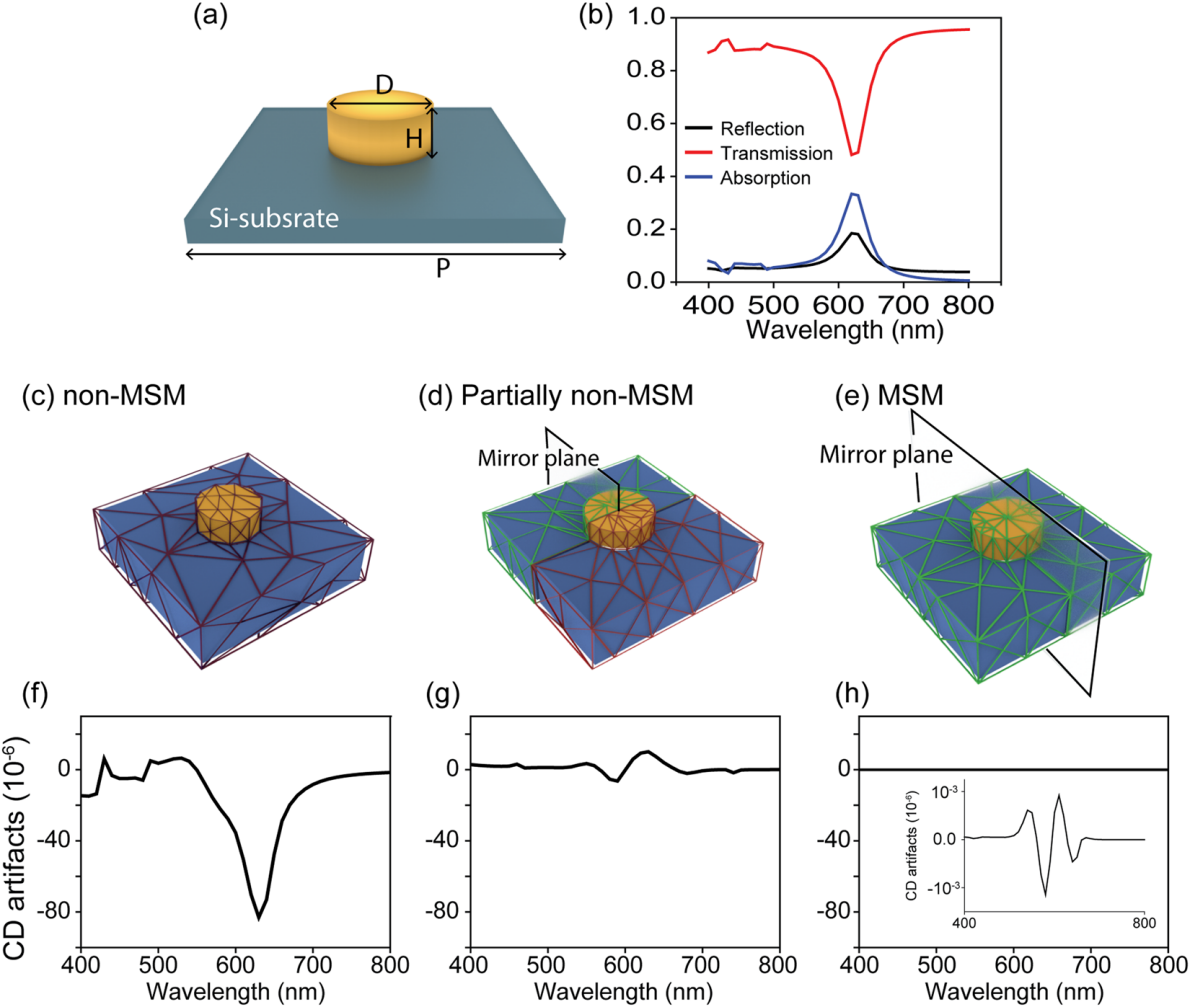
Impact of the discrete meshes on calculations of the optical responses of the gold nanodisk. (**a**) A schematic of a gold nanostructure of diameter *D* = 90 nm, height *H* = 30 nm, and lattice constant *L* = 350 nm. (**b**) Reflection, transmission, and absorption spectra of the gold nanodisk with circularly polarized incident light. Schematics of the discretization of the problem space using (**c**) non-MSM, (**d**) partially non-MSM, and (**e**) MSM schemes and their corresponding numerical artifacts, CD_mesh_, in (**f**–**h**), respectively.

Because the CD signals of chiral molecules are usually very weak^24–26^, in their case eliminating the numerical artifacts caused by mesh (CD_mesh_) is particularly important. Here, we start by demonstrating how CD_mesh_ can arise, by considering the achiral nanostructure shown in Fig. 2(a) and show the calculated artifact CD that should not exist in the system. The simulated reflection, transmission, and absorption spectra of the gold nanodisk show localized surface plasmon resonance (LSPR) at the wavelength of 620 nm. Note that, since the system is achiral, the spectra in Fig. 2(b) are independent of the polarization of the circularly-polarized incidence. Now, we calculate the CD signals for the three meshing configurations: the non-mirror symmetric mesh (non-MSM) (Fig. 2(c)), the partially non-MSM (Fig. 2(d)), and the mirror symmetric mesh (MSM) (Fig. 2(e)). The non-MSM lacks mirror symmetry, while the MSM possesses mirror symmetric geometry with respect to the mirror plane. The partially non-MSM, included to demonstrate how the meshing configuration impacts the CD_mesh_, has mirror symmetry in only half of the system. For all three configurations, we set the maximum element size of the mesh (*d*_max_) as 40 nm. As shown in Fig. 2(f,g), although in the achiral nanostructure there should be no CD signal^7^, the non-MSM and partially non-MSM configurations produce non-zero CD signals that are actually pure numerical artifacts from the mesh, CD_mesh_. We note that those CD_mesh_ are maximized at the LSPR wavelength. We also point out that CD_mesh_ from the partially non-MSM is significantly suppressed compared to that from the non-MSM. This is significant because it implies that CD_mesh_ is a numerical artifact arising from an improperly discretized problem space, and that it can be suppressed by employing the MSM configuration. As shown in Fig. 2(h), the MSM perfectly eliminates the CD_mesh_ which agrees with the expected optical response of an achiral nanostructure. The MSM almost eliminates the CD_mesh_ because it produces the same amounts of numerical artifacts from the left circular polarized (LCP) and right circular polarized (RCP) waves and so they eventually cancel each other out during the calculation of the CD, which is the difference between the LCP and RCP signals.

**Figure 3:**
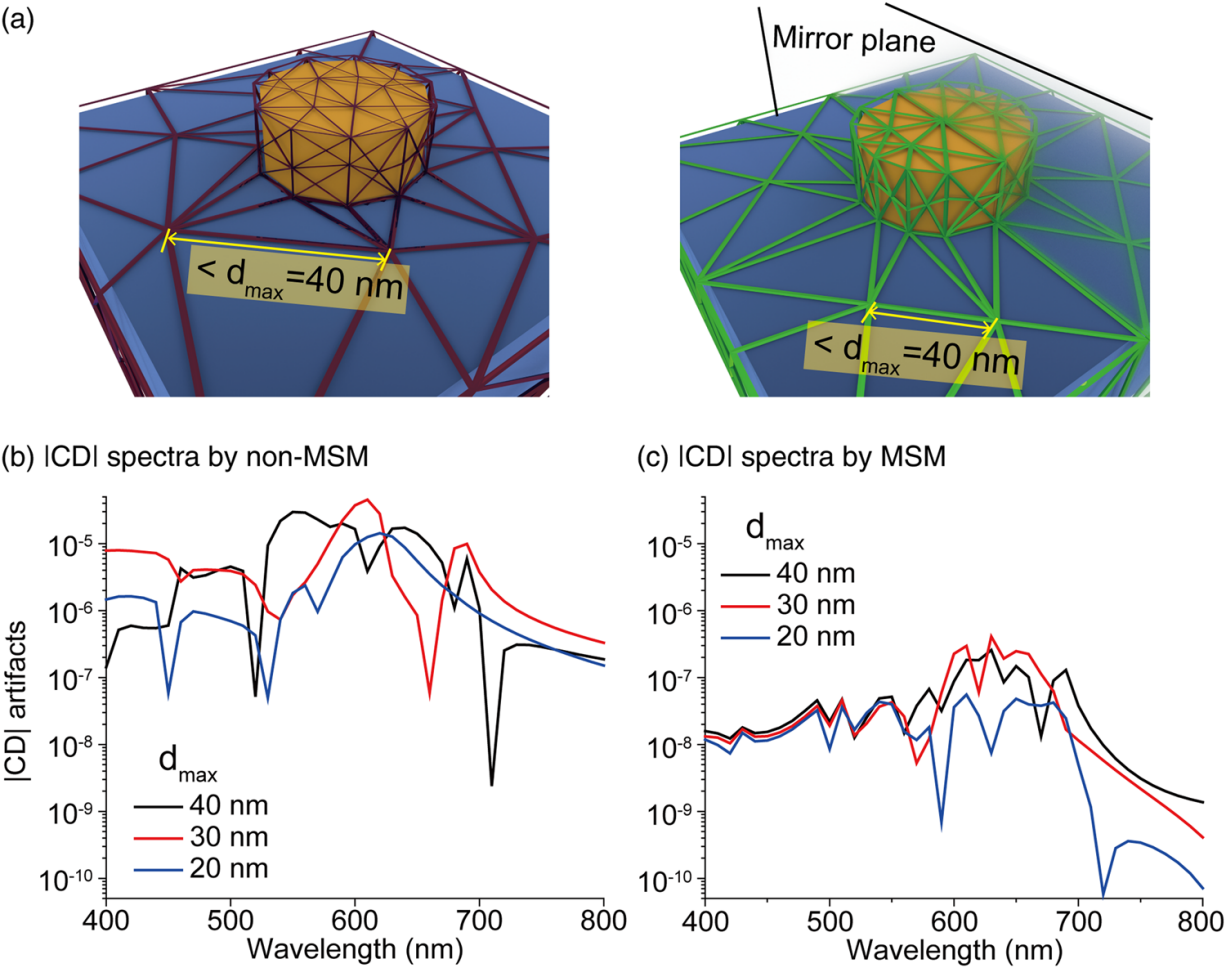
Convergence test of CD_mesh_ from non-MSM and MSM with three different maximum grid sizes. (**a**) Schematics of non-MSM and MSM. We set three maximum grid size (*d*_max_) as 40 nm, 30 nm and 20 nm. The CD_mesh_ spectra from (**b**) non-MSM and (**c**) MSM are present.

In order to see the grid-size-dependency of CD_mesh_ with non-MSM and MSM, we calculated the CD_mesh_ from the achiral nanostructure implemented with three different maximum grid sizes, d_gold_ = 40 nm, 30 nm, and 20 nm, as shown in Fig. 3(a). The spectra of CD_mesh_ from non-MSM and MSM are present in Fig. 3(b,c), respectively. We found that overall CD_mesh_ from the non-MSM and the MSM all become reduced in a convergent way as the maximum mesh size decreases. However, even the converging CD_mesh_ arising from the non-MSM is much larger than the MSM. These results confirm that, compared to non-MSM, the suppression of CD_mesh_ by MSM is quite strong even with sparse meshes, allowing much more efficient numerical calculation of CD.

### CD spectra of the chiral molecule/nanostructure coupled system using non-MSM and MSM

**Figure 4:**
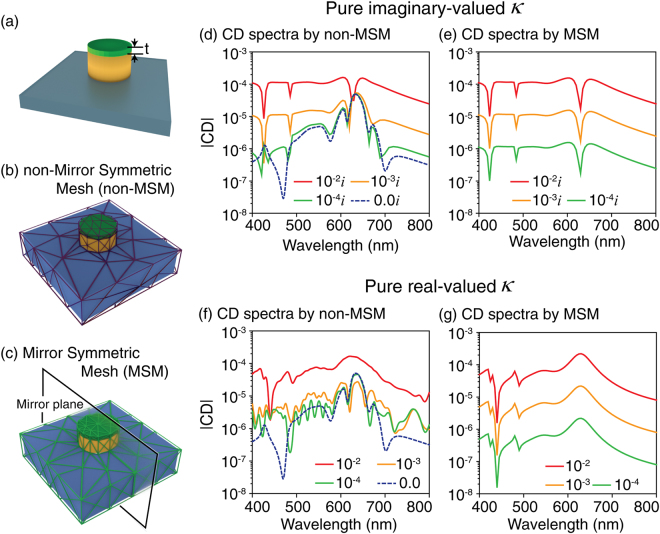
CD spectra from the chiral molecule/nanostructure coupled system with various values of chiral parameter *κ*. (**a**–**c**) Schematics of the coupled system, non-MSM and MSM. FEM-calculated CD spectra with purely imaginary-valued *κ* obtained using (**d**) MSM and (**e**) non-MSM. CD spectra with pure-real *κ* using (**f**) MSM and (**g**) non-MSM. The dotted blue curve in (**d**) is a CD spectrum with *κ* = 0, corresponding to CD_mesh_. In the calculations, the thickness of the chiral medium, *t*, is set at 5 nm.

Now, we apply our meshing scheme to the calculation of CDs by considering chiral media. As shown in Fig. 4(a), the chiral molecule which is considered as a homogenous medium of thickness *t* = 5 nm is coated onto the same nanostructure used in Fig. 2. The chiral media, which are different from the achiral media that can be considered as linear media, can be implemented by considering their constitutive relations defined as^1,2^:

1 $${\bf{D}}=\varepsilon {\varepsilon }_{0}{\bf{E}}+i\kappa {\bf{H}}/{c}_{0},$$

2 $${\bf{B}}=\mu {\mu }_{0}{\bf{H}}-i\kappa {\bf{E}}/{c}_{0}.$$

Here, *ε*_0_ and *μ*_0_ are vacuum permittivity and permeability, and *ε* and *μ* are the relative permittivity and permeability of the chiral molecule. *c*_0_ is the speed of light in free-space, and *κ*, normalized by *c*_0_, is the dimensionless chiral parameter, which describes the chiroptical response of the chiral media. We note that the real and imaginary parts of the *κ* are related to the optical rotatory dispersion and the CD, respectively.

For a better demonstration of how the numerical calculation of weak total CD signals from a chiral molecule/nanostructure coupled system can be hampered by CD_mesh_, we consider three representative values of the pure-real/imaginary chiral parameter *κ* of the molecule^24^, 0.0001, 0.001, and 0.01, to consider the cases of weak, intermediate, and strong chiral responses, respectively. Here, the maximum discrete grid size is set at 40 nm for all FEM simulations. Figure 4(d,e) show FEM-calculated CD spectra considering the three pure-imaginary *κ* by adopting non-MSM and MSM. As shown in Fig. 4(e), the CD spectra by MSM exhibit identical line-shapes for the given values of *κ*. However, Fig. 4(d) clearly shows that FEM by non-MSM does not provide accurate CD values, as the CD spectra strongly differ from those by MSM and there is no consistency of spectral line-shape with varying *κ*. In particular, note that the discrepancies between the results obtained with MSM and non-MSM become more pronounced as *κ* decreases. The origin of the discrepancy is CD_mesh_, the dotted-curve in Fig. 4(d), which is obtained by turning off the chiral parameter (*κ* = 0). CD_mesh_ is not negligible since its magnitude is comparable to those of the *pure* CDs obtained by applying MSM to the weak and intermediate cases (*κ* = 0.001*i* and 0.0001*i*). Similarly, in the case of the purely real-valued *κ* shown in Fig. 4(f,g), non-MSM does not provide reliable CD results while MSM generates consistently identical CD line-shapes. Again, the discrepancy between the results from MSM and non-MSM is more pronounced with smaller *κ*. Therefore, suppressing the numerical artifact is particularly important in the calculation of CDs from chiral molecules that exhibit weak chiral responses.

**Figure 5:**
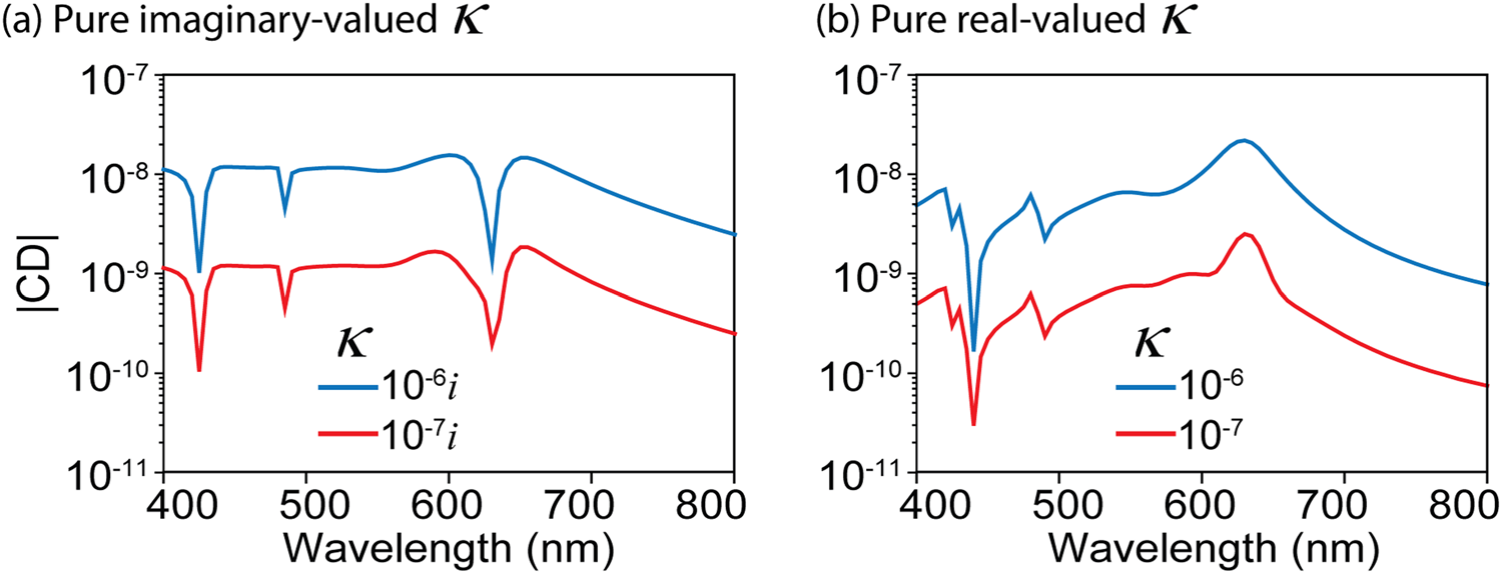
CD spectra with extremely small *κ* calculated by applying MSM. CD spectra with (**a**) pure-imaginary and (**b**) pure real *κ*.

We further applied our MSM to the calculation of extremely weak CD signals. Figure 5 shows the CD signals of the chiral molecule/nanostructure coupled system with an even smaller *κ*. For both pure-imaginary (Fig. 5(a)) and pure-real (Fig. 5(b)) *κ*, MSM gives consistent CD spectra even at the low magnitude of 10^−10^, far-lower than that of the CD_mesh_. This suggests that our MSM enables numerical calculation of extremely weak CD signals, such as the calculation of CD from a system featuring dilute chiral molecule solutions.

**Figure 6:**
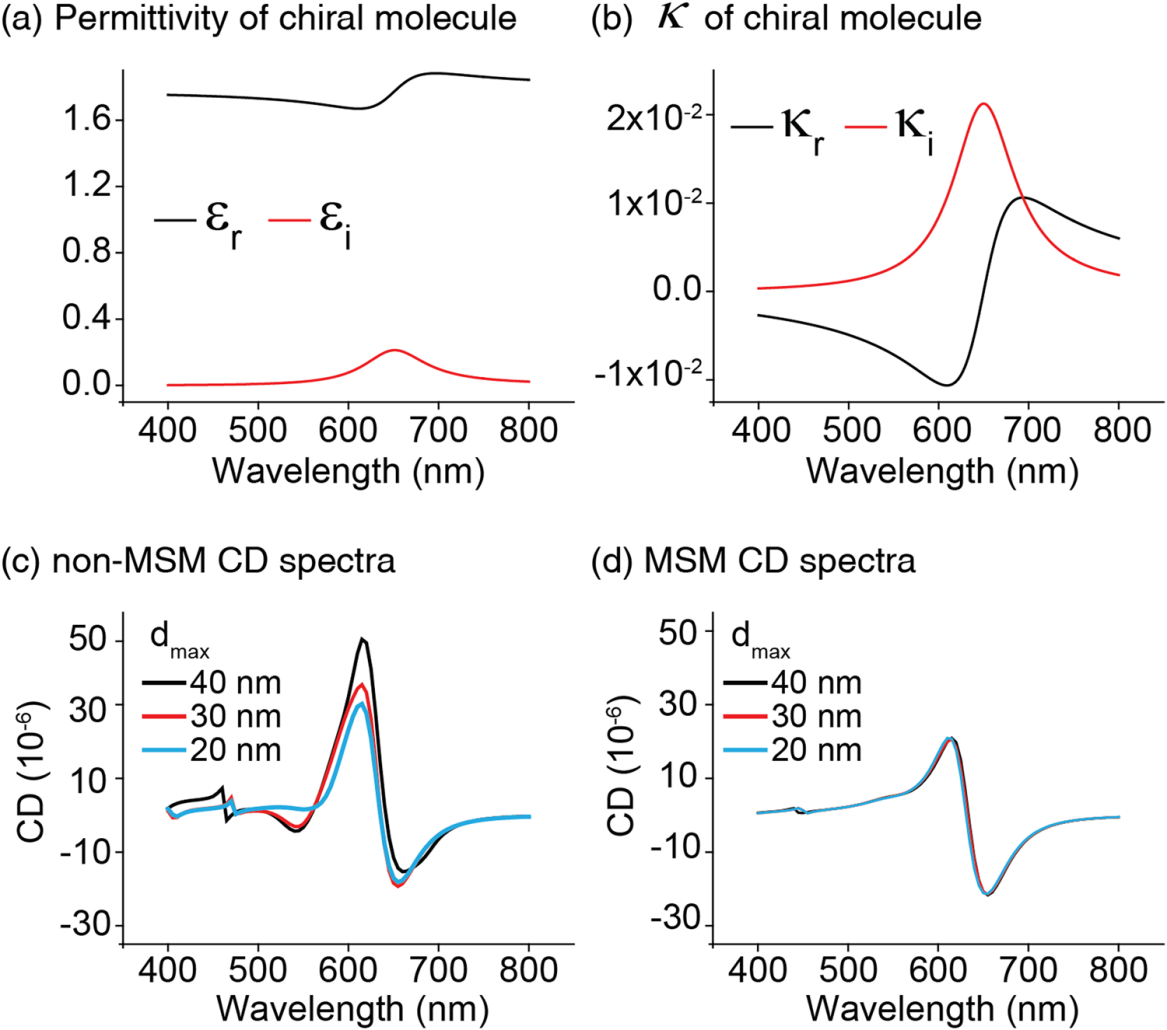
Numerical convergence test of CD spectra with decreasing maximum finite element size. (**a**,**b**) Assumed frequency dependence of the dielectric constant and chiral parameter *κ* of the chiral molecules. CD spectra obtained by (**c**) non-MSM and (**d**) MSM with various maximum grid sizes. In (**d**), MSM gives almost identical CD spectra regardless of the maximum grid size, while in (**c**) non-MSM yields spectra that are not fully saturated. In the calculations, we set *t* = 3 nm.

In principle, because CD_mesh_ is a numerical artifact related to the discretization of space, it can be suppressed by implementing smaller finite elements. This scheme is often used in the mesh convergence test which is an important procedure for the quantitative verification of numerical results^27^. To check both the quantitative accuracy of results from MSM and for the possible suppression of CD_mesh_ in non-MSM, the chiral molecule/nanostructure coupled system is meshed with three different maximum-grid-sizes, *d*_max_. Also, in order to take into account the more general chiral response of the molecule, we considered the complex dielectric constant and the chiral parameter as shown in Fig. 6(a,b), respectively: they are assumed to follow the Lorentz and Condon models. The resonance wavelength of the chiral molecules is intentionally set to be the same wavelength as LSPR to mimic the chiral response boost by the nanostructure. The FEM-calculated CD signals under the non-MSM and MSM schemes with three *d*_max_ values are shown in Fig. 6(c,d). It is clearly shown that decreasing *d*_max_ suppresses CD_max_ in the non-MSM case. However, unlike the MSM case in which the CD spectra are almost identical regardless of *d*_max_, in the non-MSM case even reducing *d*_max_ from 40 nm to 20 nm does not yield fully-saturated results, since at 20 nm *d*_max_ an appreciable discrepancy still exists between the non-MSM and MSM CD spectra. Although further reductions in *d*_max_ can be expected to produce more accurate results when using non-MSM, it should be noted that the size of the discrete grid is the main factor that trades off numerical accuracy for computational complexity. The results in Fig. 6 summarize that our MSM has a high tolerance for larger grid sizes, enabling computationally efficient and quantitatively accurate CD calculations.

### Checking the numerical validity of the implementation of chiral media

**Figure 7:**
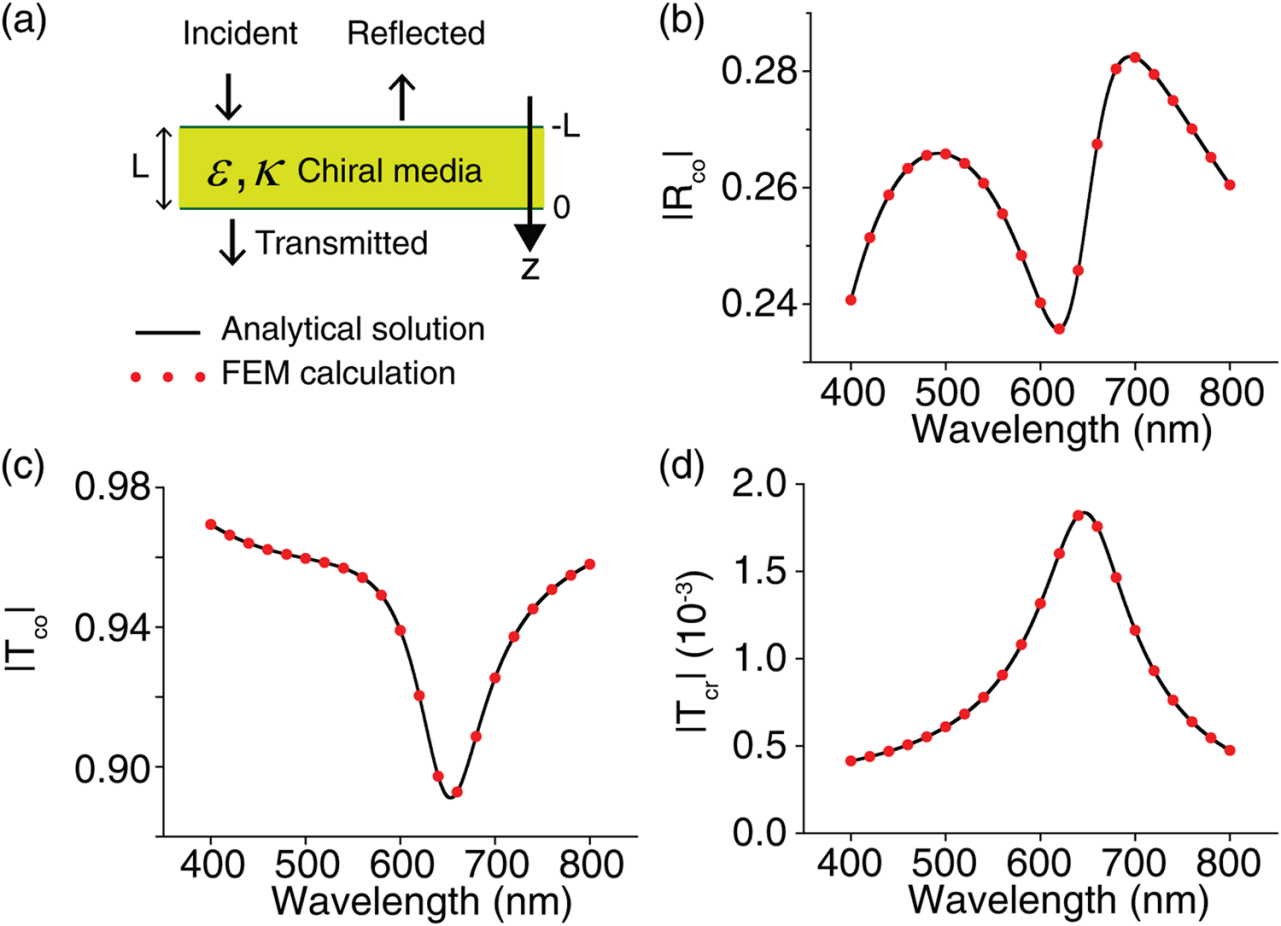
Validity of the numerical implementation of the chiral media in FEM. (**a**) A schematic of a free-standing chiral slab of thickness *L* = 100 nm. The frequency dependent dielectric constant and chiral parameter *κ* of the slab are given in Fig. 6(a,b), respectively. FEM-calculated (red dots) and analytically calculated (solid lines) spectra of (**b**) co-polarized reflection *|R*_*co*_*|*, (**c**) co-polarized transmission *|T*_*co*_*|*, and (**d**) cross-polarized transmission *|T*_*cr*_*|*.

The numerical validity of the implementation of chiral media is checked by comparing the numerically and analytically calculated chiral responses of the chiral molecule slab shown in Fig. 7(a). We used normally incident linearly polarized light, propagating along the z-direction. We compared three coefficients of the chiral slab: co-reflection *R*_*co*_, co-transmission *T*_*co*_, and cross-transmission *T*_*cr*_, which can be calculated as^2^

3 $${R}_{co}=\frac{({\eta }_{1}{\eta }_{2}-{\eta }_{2}{\eta }_{3})\cos ({k}_{2}L)-i({\eta }_{1}{\eta }_{3}-{\eta }_{2}^{2})\sin ({k}_{2}L)}{({\eta }_{1}{\eta }_{2}+{\eta }_{2}{\eta }_{3})\cos ({k}_{2}L)-i({\eta }_{1}{\eta }_{3}+{\eta }_{2}^{2})\sin ({k}_{2}L)},$$

4 $${T}_{co}=\frac{2{\eta }_{2}{\eta }_{3}\,\cos ({\kappa }_{r}{k}_{2}L)}{({\eta }_{1}{\eta }_{2}+{\eta }_{2}{\eta }_{3})\cos ({k}_{2}L)-i({\eta }_{1}{\eta }_{3}+{\eta }_{2}^{2})\sin ({k}_{2}L)},$$

5 $${T}_{cr}=\frac{-2{\eta }_{2}{\eta }_{3}\,\sin ({\kappa }_{r}{k}_{2}L)}{({\eta }_{1}{\eta }_{2}+{\eta }_{2}{\eta }_{3})\cos ({k}_{2}L)-i({\eta }_{1}{\eta }_{3}+{\eta }_{2}^{2})\sin ({k}_{2}L)},$$

where *k*_2_ is the wavenumber of light in the chiral slab, and *L* is the thickness of the slab. Note that the cross-reflection *R*_*cr*_ is zero for all values of *κ*. We assume that the chiral slab is freestanding, so that impedances of the incident region (−*L* < *z* < 0) *η*_1_ and the transmitted region (0 < *z*) *η*_3_ become the impedance of the vacuum: ${\eta }_{1}={\eta }_{3}={\eta }_{0}=\sqrt{{\mu }_{0}/{\varepsilon }_{0}}$. We also assumed that the slab possesses the exemplary dielectric constant and chiral parameter shown in Fig. 6(a,b), respectively. Results from the FEM calculation and analytic model are shown in Fig. 7(b–d). All the numerical results perfectly agree with the analytical theory, confirming that chiral media are well implemented by our FEM simulation.

## Discussion

**Figure 8:**
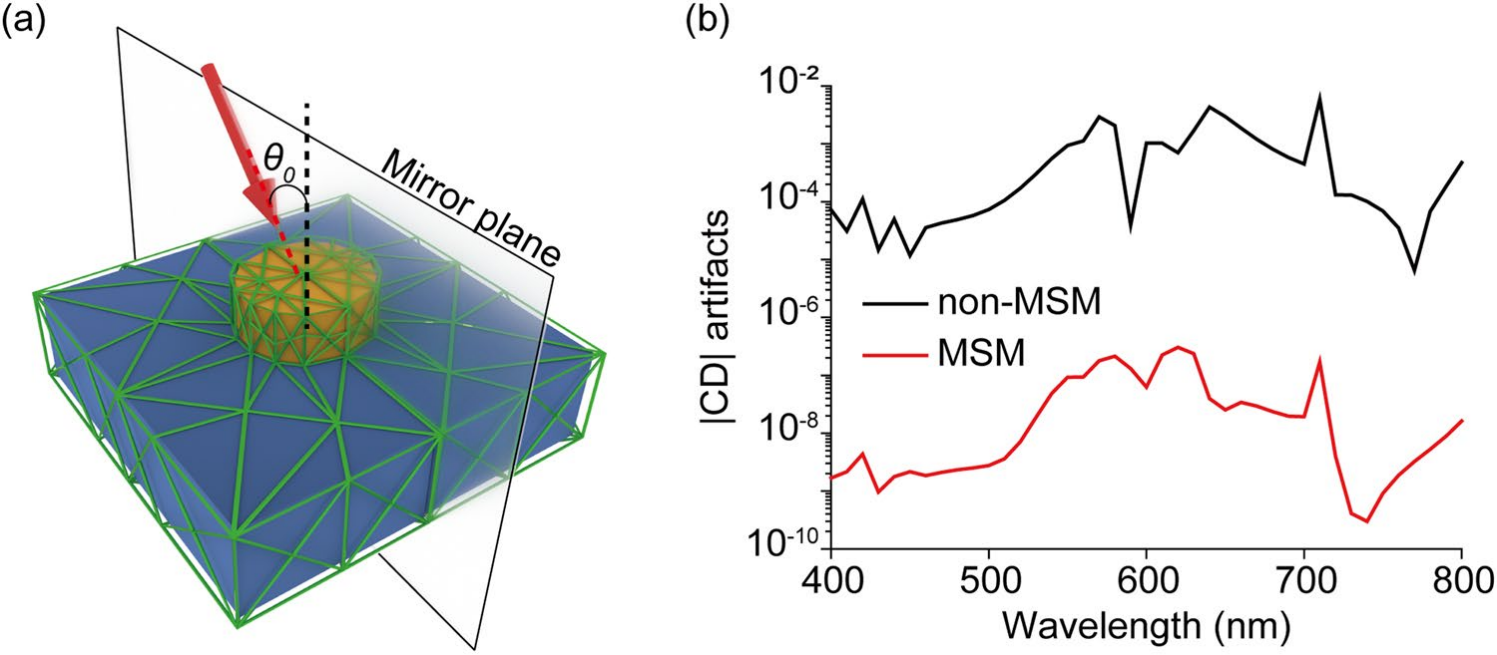
MSM for non-normal incidence case. (**a**) Non-normally incident light (*θ*_0_ = 45°) to the periodic nanodisk array. (**b**) CD_mesh_ spectra of the nanodisk. CD should be zero since the nanodisk is achiral.

So far, we have considered only the case of normal incidence. We note that our mirror symmetric meshing (MSM) scheme can be extended to the case of non-normal incidence by taking the mirror plane to contain the light wavevector. For an exemplary demonstration, we studied the optical response of a periodic nanodisk array to an incoming light with incidence angle *θ*_0_ = 45° with respect to the vertical axis of the simulation domain, as shown in Fig. 8(a). Explicit calculation, with results in Fig. 8(b), shows that CD_mesh_ of non-MSM is about thousand-times larger than that of MSM with the mirror plane shown in Fig. 8(a).

**Figure 9:**
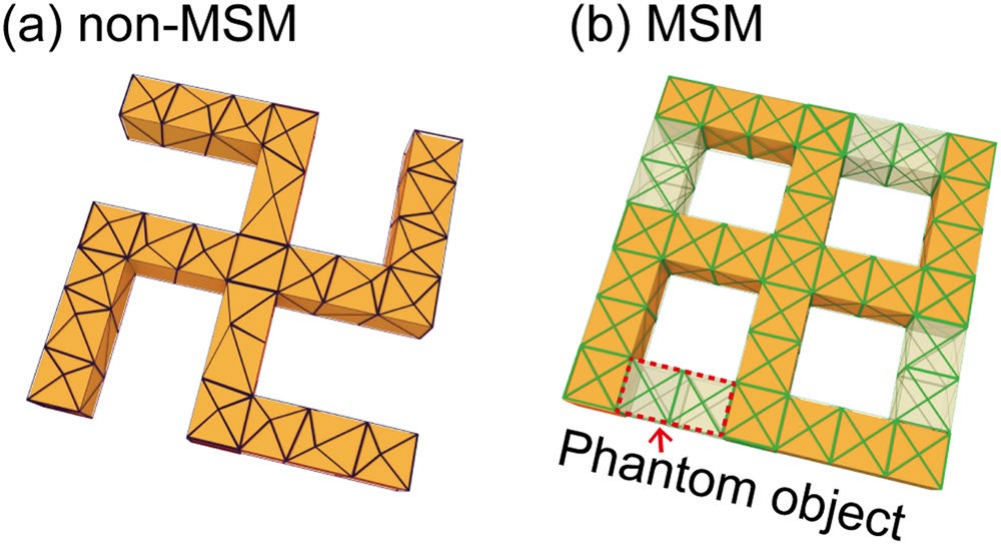
Implementation of MSM for chirally-arranged nanostructure. (**a**) An exemplary structure of a gammadion metamaterial. (**b**) MSM implemented by introducing phantom object.

We also note that our MSM scheme can be extended to the case of chiral structures made of non-chiral materials. In order to construct MSM for systems without mirror-symmetry, we introduce phantom objects to restore the mirror symmetry and create mirror-symmetric mesh without actually changing the real structure. Figure 9(a) presents an exemplary chiral gammadion structure. We change the gammadion into an achiral structure by minimally adding phantom object and create MSM and then remove the phantom part. The resulting mesh is given in Fig. 9(b) and explicit calculation shows significantly reduced CD_mesh_.

In conclusion, we introduced a mirror symmetric mesh (MSM) scheme that can be applied to the finite element method (FEM) for numerical calculations of circular dichroism (CD). We demonstrated that unwanted numerical artifacts in CD, arising from improperly discretized problem spaces, can be strongly suppressed by adopting MSM. Our MSM scheme was tested through the calculation of CD from a chiral molecule/nanodisk coupled system, and we showed that MSM exhibits much faster numerical convergence and superior numerical reliability than the usual meshing scheme, enabling efficient and accurate CD calculations.
